# Optic Neuritis Is Associated with Inner Nuclear Layer Thickening and Microcystic Macular Edema Independently of Multiple Sclerosis

**DOI:** 10.1371/journal.pone.0071145

**Published:** 2013-08-06

**Authors:** Falko Kaufhold, Hanna Zimmermann, Elisa Schneider, Klemens Ruprecht, Friedemann Paul, Timm Oberwahrenbrock, Alexander U. Brandt

**Affiliations:** 1 NeuroCure Clinical Research Center and Experimental and Clinical Research Center, Charité – Universitätsmedizin Berlin and Max Delbrück Center for Molecular Medicine, Berlin, Germany; 2 Clinical and Experimental Multiple Sclerosis Research Center, Department of Neurology, Charité – Universitätsmedizin Berlin, Berlin, Germany; University of Jaén, Spain

## Abstract

**Background:**

Microcystic macular edema (MME) and inner nuclear layer thickening (INL) were described in multiple sclerosis (MS) and neuromyelitis optica (NMO) patients using optical coherence tomography (OCT). The cause of these findings is currently unknown and a relation to inflammatory or degenerative processes in the optic nerve is discussed.

**Objective:**

The aim of our study was to investigate whether INL thickening and MME are related to optic neuritis (ON) in various neuro-inflammatory disorders causingON: MS, NMO and chronic inflammatory optic neuropathy.

**Methods:**

We retrospectively analyzed data from 216 MS patients, 39 patients with a clinically isolated syndrome, 20 NMO spectrum disorder patients, 9 patients with chronic inflammatory optic neuropathy and 121 healthy subjects. Intra-retinal layer segmentation was performed for the eyes of patients with unilateral ON. Scanning laser ophthalmoscopy (SLO) images were reviewed for characteristic ocular fundus changes.

**Results:**

Intra-retinal layer segmentation showed that eyes with a history of ON displayed MME independent INL thickening compared to contralateral eyes without previous ON. MME was detected in 22 eyes from 15 patients (5.3% of all screened patients), including 7 patients with bilateral edema. Of these, 21 had a prior history of ON (95%). The SLO images of all 22 MME-affected eyes showed crescent-shaped texture changes which were visible in the perifoveal region. A second grader who was blinded to the results of the OCT classified all SLO images for the presence of these characteristic fundus changes. All MME eyes were correctly classified (sensitivity = 100%) with high specificity (95.2%).

**Conclusion:**

This study shows that both MME and INL thickening occur in various neuro-inflammatory disorders associated with ON. We also demonstrate that detection and analysis of MME by OCT is not limited to B-scans, but also possible using SLO images.

## Introduction

Optic neuritis (ON) is a common symptom of inflammatory central nervous system disorders that often heralds a diagnosis of multiple sclerosis (MS) or neuromyelitis optica (NMO) [1]–[5]. In both diseases, ON can cause irreversible damage to axons in the retina and their neurons, the retinal ganglion cells, which can be quantified by optical coherence tomography (OCT) [6]–[10]. A number of OCT studies have demonstrated retinal neuro-axonal damage in MS patients with and without a history of ON, as well as in NMO patients [7], [8], [11]–[18]. Thanks to recent technical advances in OCT, including improved resolution and intra-retinal layer segmentation, pathologies can be detected in various retinal layers, such as the retinal ganglion cell layer (GCL) and the inner nuclear layer (INL) [19]–[24]. The new data this has yielded have substantially advanced our understanding of retinal pathology in MS, leading to intriguing, new hypotheses regarding MS pathophysiology [19].

Recently, a retinal finding termed microcystic macular edema (MME) was observed in MS [25], [26]. While this paper was under review, two further studies reported MME in NMO [27], [28]. In all studies, MME was predominantly located in the INL and was associated with reduced visual acuity and retinal nerve fiber layer (RNFL) thinning in both disorders. In MS, the INL was shown as a prominent site of retinal and microglial inflammation [29], which led the authors to propose MME as a clinical correlate of pathological processes in the INL. Indeed, an earlier study had already reported an correlation between INL thickening at baseline with more severe disease progression, as measured by increased development of contrast-enhancing lesions, new T2 lesions, Extended Disability Status Scale (EDSS) [30] progression and relapses in patients with relapsing-remitting MS over the study period [26]. However, other reports have countered these findings, arguing that MME may not be MS- or NMO-specific [31] or even that it is entirely independent of inflammation and instead based on acute optic neuropathy e.g. mediated by mechanical stress [32], [33].

Against this background, our first aim was determining whether INL thickening only occurs in MME and whether it is related to a previous episode of ON, which would suggest that ON is as a causative factor in INL pathology. We screened patients with three different neuro-inflammatory disorders including ON: MS, NMO and chronic relapsing inflammatory optic neuropathy (CRION) [34]. Secondly, we report that MME can be detected and quantified more easily in fundoscopic scanning laser ophthalmoscopy (SLO) images than on OCT B-scans, which has the potential to make screening and analysis of MME in research and clinical routine more robust.

## Methods

### Patients

Scans acquired between June 2010 and August 2012 archived in the NeuroCure Clinical Research Center’s OCT database were screened for MME. The study included 216 MS patients diagnosed according to the revised 2005 McDonald criteria [35], 20 NMO-spectrum disorder (NMOSD) patients diagnosed according to current diagnostic criteria [36], [37], 39 patients with clinically isolated syndrome (CIS), as identified by application of the revised McDonald criteria [38], 9 patients diagnosed with CRION and 121 healthy subjects. All NMOSD patients were tested for the presence of antibodies against aquaporin-4 (AQP4) in at least one of several assays, of which 16 tested positive (80%). Some ophthalmologic features of 17 patients from the NMOSD cohort have been previously reported by Schneider et al. [39].

Inclusion criteria were a history of ON or lack of ON clearly confirmed by medical records. In contrast, exclusion criteria were other neurological, ophthalmological and systemic diseases that damage the optic nerve or retina (i.e. glaucoma, diabetes, age-related macular degeneration, epiretinal membranes) or a history of fingolimod treatment, which is suspected to cause macular edema [40], [41]. Clinical data, including disease duration and current medical treatment, were compiled as part of a comprehensive neurological examination under the supervision of a board-certified neurologist. MS patients were classified for MS subtypes according to the Lublin criteria [42]. Neurological disability was assessed using EDSS [30] and the Global Multiple Sclerosis Severity Score (MSSS) [43]. Finally, the high-contrast visual acuity of all subjects was quantified using Snellen charts or ETDRS charts [44].

### Ethics Statement

The study was approved by the local ethics committee of the Charité – Universitätsmedizin Berlin and was conducted in accordance with the Declaration of Helsinki in its current version, the guidelines of the International Conference on Harmonisation of Good Clinical Practice (ICH-GCP) and applicable German laws. All participants gave informed, written consent.

### Optical Coherence Tomography

Peripapillary RNFL thickness (pRNFL) and macular volume scans were performed using a spectral-domain OCT (Heidelberg Spectralis, Heidelberg Engineering, Germany, Heidelberg Eye Explorer viewing module versions 5.2.4.0–5.6.1.0) of each patient or healthy subject’s eyes. pRNFL thickness was measured with a 12° circular scan (approx. 3.4 mm diameter) around the optic nerve head obtained using the device’s standard protocol and segmentation algorithm with activated eye tracker. Whenever possible, the maximum number of averaging frames in the automatic-real-time mode (ART) was used. Macular volume was measured using a custom protocol that generated 61 vertical slices (B-scans) focusing the fovea, at scanning angle of 30°×25° and a resolution of 768 A-scans per B-scan. As this scan protocol limits averaging frames to 13, the sensitivity should be sufficient to detect MME in B-scans, which might be undetectable at higher ART settings [25]. Total macular volume (TMV) was calculated by estimating the distance between the inner limiting membrane and Bruch’s membrane in a 6 mm-diameter cylinder using the OCT software’s segmentation algorithm. All scans were acquired by experienced operators and were evaluated for sufficient signal strength, correct centering and segmentation based on the OSCAR-IB criteria by a second operator [45]. In total, 12 eyes of 12 different subjects (5 MS; 1 NMOSD; 6 healthy controls) had no usable OCT scan (neither RNFL circle nor macular volume scan) and were completely excluded from analysis for one of the following reasons: the RNFL scans of two eyes were truncated, one fundus image was weakly illuminated and another RNFL scan was not correctly centered and no eye-tracker had been used. The remaining RNFL and all macular volume scans had not been performed or archived because of lack of patient compliance or, in case of healthy controls, due to time constraints.

To identify eyes with MME, one operator applied the criteria published by Gelfand and colleagues [25] and reviewed all macular scans according to these criteria. MME was defined as clearly limited, insular and cystoid areas of hyporeflectivity in two or more consecutive B-scans. Shadowing in the retinal layers below the cystoid abnormalities was a second but optional criterion for MME inclusion. Blood vessels shadows were excluded as MME criterion.

Furthermore, we investigated the influence of MME and previous ON on the thickness of the inner retinal layers. Retinal layer segmentation was performed for patients with MME (n = 15) or with a previous unilateral ON event (excluding the patients with MME) (n = 75) and for 39 healthy controls. Controls were age- (±3 years) and gender-matched to patients with unilateral ON without MME and were chosen from all controls using the optmatch R package. Here, for each eye, the central B-scan through the fovea and every fourth B-scan in temporal and nasal direction were automatically segmented (Heidelberg Software 1.7.1.0), reviewed and manually revised by two graders to correct any overt segmentation errors. Both graders were blinded as to subject identity and clinical status (ON or eyes without a history of ON (NON)). In addition, graders were not aware which eyes were belonging together but in all cases, one and the same grader corrected both eyes of an individual subject The average layer thicknesses of the macular RNFL (mRNFL), the combined ganglion cell and inner plexiform layer (GCIPL) and the INL were assessed for the area calculated in the earlier TMV estimation. Grader consistency was estimated using 10 randomly selected patient eyes (all MS) each corrected by both graders. Intraclass correlation coefficients were 0.997 for the mRNFL and 0.999 for both the GCIPL and INL.

### MME Detection in OCT SLO Images

**Figure 1:**
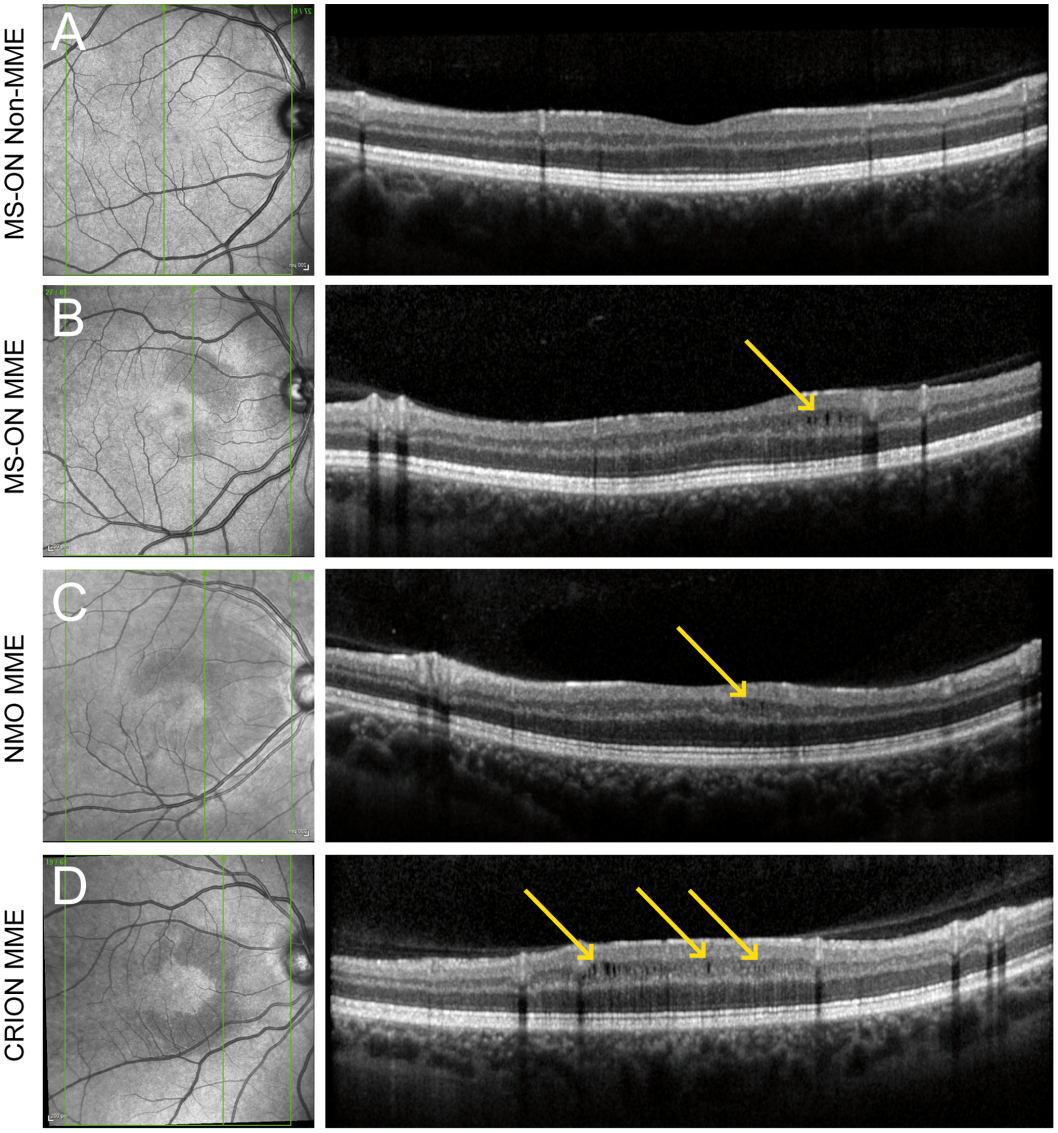
Sample scans showing MME in SLO and OCT images. Sample SLO images (on the left) and sample OCT B-scan images (on the right) from A) MS patient’s eye with a history of ON but without MME, B) an MS patient’s eye with history of ON and MME, C) an NMO patient’s eye with history of ON and MME and D) an eye from a patient with CRION and MME. Whereas the eye from A does not show any signs of MME in either the SLO or OCT B-scan image, all eyes in B-D show similar findings. The SLO images were mirrored where necessary to standardize orientation.

While screening OCT B-scans for MME the operator (FK) detected distinct changes of the ocular fundus in the SLO-images from eyes diagnosed with MME using the Spectralis built-in confocal SLO (wavelength = 820 nm). In a second step, an independent reviewer (HZ) familiar with the SLO findings from a sample SLO image but blinded to the MME results from OCT scans screened all SLO images from the database. MME was considered present, if the SLO images showed: (i) a darker and dot-like pattern in the macular area compared to the surrounding tissue, and (ii) crescent-like configurations spanning parts of the macula around the fovea (see Results and Figure 1). A third operator (TO) quantified the affected SLO areas using ImageJ (version 1.44). A rigorous blinding procedure was followed to ensure classification by the three operators remained independent. All operators were blinded for age, gender, disease and ON history. All SLO-image evaluators were blinded to the corresponding OCT images and to the result of MME OCT screening.

### Statistical Analysis

Comparison of age and EDSS between cohorts was performed using the Mann-Whitney-U test. Gender differences were assessed with Pearson’s chi-squared test. Association between SLO area size and visual acuity, time since last ON, pRNFL thickness and TMV were analyzed using generalized estimating equation models (GEE) to account for inter-eye/intra-patient dependencies using the working correlation matrix “exchangeable”. In all GEE models, the SLO area was set as dependent variable. Likewise, group differences in visual acuity and OCT results were calculated using GEE models. Provided parameters from GEE models are effect size (B), standard error (SE) and significance (P). Frequency of MME occurrence was compared between the cohorts using Fisher’s exact tests. Comparison of intra-patient/inter-eye differences was performed using the two-sided paired Wilcoxon signed rank test. Correlation of mRNFL and GCIPL differences to the INL differences was analyzed using linear regression models. Statistical tests were performed with IBM SPSS 20 (IBM, Armonk, NY, USA) or R (basic package ver. 2.15.2, including the packages: geepack (ver. 1.1.6), ggplot2 (ver. 0.9.3) and optmatch (ver. 0.8.1)). Statistical significance was established at *P*<0.05. As ours was an exploratory study, no power calculation had previously been performed, and the disorders of all included groups are related and their features therefore likely correlate, no correction for multiple comparisons was performed.

## Results

Based on the criteria defined by Gelfand and colleagues [25], we identified MME in 22 eyes from 15 patients. Of these patients, 5 had been diagnosed with CRION, 3 with NMOSD and 7 with MS. No MME was observed in healthy controls and the screened CIS patients. A demographic overview of the study cohort is presented in Table 1. MME appeared bilaterally in 7 of the 15 patients and was located exclusively in the inner nuclear layer of the retina, between 925 µm and 2,200 µm (mean ± SD: 1,602±351 µm) distal to the foveal center. The maximum cyst diameter was 81 µm (mean ± SD: 45±14 µm).

**Table 1:** Demographic overview of the study cohort.

	Parameter	MME patients	Non-MME patients	HC
Subjects	Total (N)	15	268	121
	MS (N)	7	209	–
	CIS (N)	0	38	–
	NMO (N)	3	17	–
	CRION (N)	5	4	–
Age (years)	Mean ± SD	40±10	42±11	36±12
	Min – Max	23–54	17–72	20–68
Gender (female/male)	N/N	12/3	171/97	83/38
Disease Duration (months)	Mean ± SD	125±111	83±79	–
	Min – Max	10–317	0–403	–
MS-Subtypes	RRMS (N)	3	163	–
	SPMS (N)	4	31	–
	PPMS (N)	0	15	–

**Abbreviations:** MME = microcystic macular edema; MS = multiple sclerosis; NMO = neuromyelitis optica spectrum diseases; CRION = chronic relapsing inflammatory optic neuropathy; HC = healthy control; RRMS = relapsing-remitting multiple sclerosis; SPMS = secondary-progressive multiple sclerosis; PPMS = primary-progressive multiple sclerosis; CIS = Clinical isolated syndrome; SD = standard deviation; Min = minimum value; Max = maximum value.

### MME is Associated with ON

A prior history of ON was found in all MME-affected patient eyes, with one exception (95.5%). In the latter case, the patient had been diagnosed with secondary progressive MS and reported experiencing visual disturbances in the eye during the 1980s. The most recent documented VEP from this eye had a latency of 116 ms. Eyes with MME showed reduced visual acuity (*P* = 0.010), along with decreased pRNFL thickness (*P*<0.001) and TMV (*P*<0.001), compared to eyes unaffected by MME. A summary of the key ocular data of the MME and non-MME eyes, including ON prevalence, visual acuity, pRNFL thickness and TMV measures, is given in Table 2.

**Table 2:** Ocular key data of MME and non-MME eyes.

	Parameter	MME eyes	Non-MME eyes a)	HC eyes
Number of eyes	Total (N)	22	538	236
	MS (N)	11	416	–
	CIS (N)	0	76	–
	NMO (N)	4	35	–
	CRION (N)	7	11	–
ON prevalence	Total N (%)	21 (95.5)	186 (34.6)	–
	MS N (%)	10 (90.9)	150 (36.1)	–
	CIS N (%)	–	15 (19.7)	–
	NMO N (%)	4 (100)	15 (42.9)	–
	CRION N (%)	7 (100)	6 (54.5)	–
Time since last ON (months)	Mean ± SD	100±100	–	–
	Min – Max	1–304	–	–
Visual acuity	Mean ± SD	0.31±0.38	1.05±0.37	1.17±0.39
	Min – Max	0–1.25	0.01–1.6	0.13–1.6
pRNFL thickness (µm)	Mean ± SD	51.6±12.5	87.5±15.65	98.4±8.49
	Min – Max	31.8–78.2	29.1–129.9	79.9–120.0
TMV (mm^3^)	Mean ± SD	8.00±0.41	8.40±0.44	8.67±0.33
	Min – Max	7.23–8.78	7.06–9.37	7.72–9.47

a) The contralateral eyes of unilateral MME eyes were excluded.

**Abbreviations:** MME = microcystic macular edema; MS = multiple sclerosis; NMO = neuromyelitis optica spectrum diseases; CRION = chronic relapsing inflammatory optic neuropathy; ON = optic neuritis; pRNFL = peripapillary retinal nerve fiber layer; TMV = total macular volume; SD = standard deviation; Min = minimum value; Max = maximum value.

### Frequency of MME in ON Eyes

In MS patients, 6.3% of all eyes with a history of ON showed MME, compared to 21.0% of all NMOSD patients’ eyes with a history of ON. MME prevalence was highest in CRION patients at 53.8% of all eyes of patients with a history of ON. The prevalence of MME was significantly different between MS and CRION patients (*P*<0.001), but not between MS and NMOSD patients (*P* = 0.068), or between NMOSD and CRION patients (*P* = 0.295).

### ON Eyes with MME are More Severely Affected

In eyes with a history of ON, MME-affected eyes showed significantly reduced pRNFL thickness (mean ± SD: 77.5±15.8 µm, *P*<0.001) and visual acuity (mean ± SD: 1.0±0.39, *P* = 0.049) and non-significantly reduced TMV (mean ± SD: 8.15±0.43 mm^3^, *P* = 0.155), compared to eyes unaffected by MME.

Eyes with pRNFL below the first quartile of all ON eyes (Q_1_ = 64.6 µm) were categorized as having a severe history of ON, which was a strong predictor for the development of MME (odds ratio = 13.6, 95% CI = 4.7–39.7).

### Disease Severity in MS-ON Patients with and without MME

MS patients with MME were later in the disease course with a higher rate of secondary progressive patients (*P* = 0.01) and had significantly higher EDSS and MSSS scores (Table 3), compared to MS patients with a history of ON but no MME.

**Table 3:** Disease severity in MS patients.

		MME MS patients	Non-MME MS-ON patients	P-value
Subjects	N	7	100	
MS subtype	RRMS (N)/SPMS (N)	3/4	87/13	0.012
EDSS	Median	5	2.25	0.002
	Min - Max	2.5–6.5	0–6.5	
Disease duration (months)	Mean ± SD	172.14±109.44,	114.38±83.59,	0.174
	Min - Max	45–303	2–403	
MSSS (mean +- SD, min - max)	Mean ± SD	5.99±1.88	3.81±2.06	0.011
	Min - Max	3.69 8.83	0.22–8.3	

**Abbreviations:** MME = microcystic macular edema; MS = multiple sclerosis ON = optic neuritis; SD = standard deviation; Min = minimum value; Max = maximum value; EDSS = Expanded Disability Status Scale; MSSS = Multiple Sclerosis Severity Score.

### Comparison of Retinal Layer Thickness between Patient Eyes with MME, Unilateral History of Optic Neuritis and Healthy Controls

**Figure 2:**
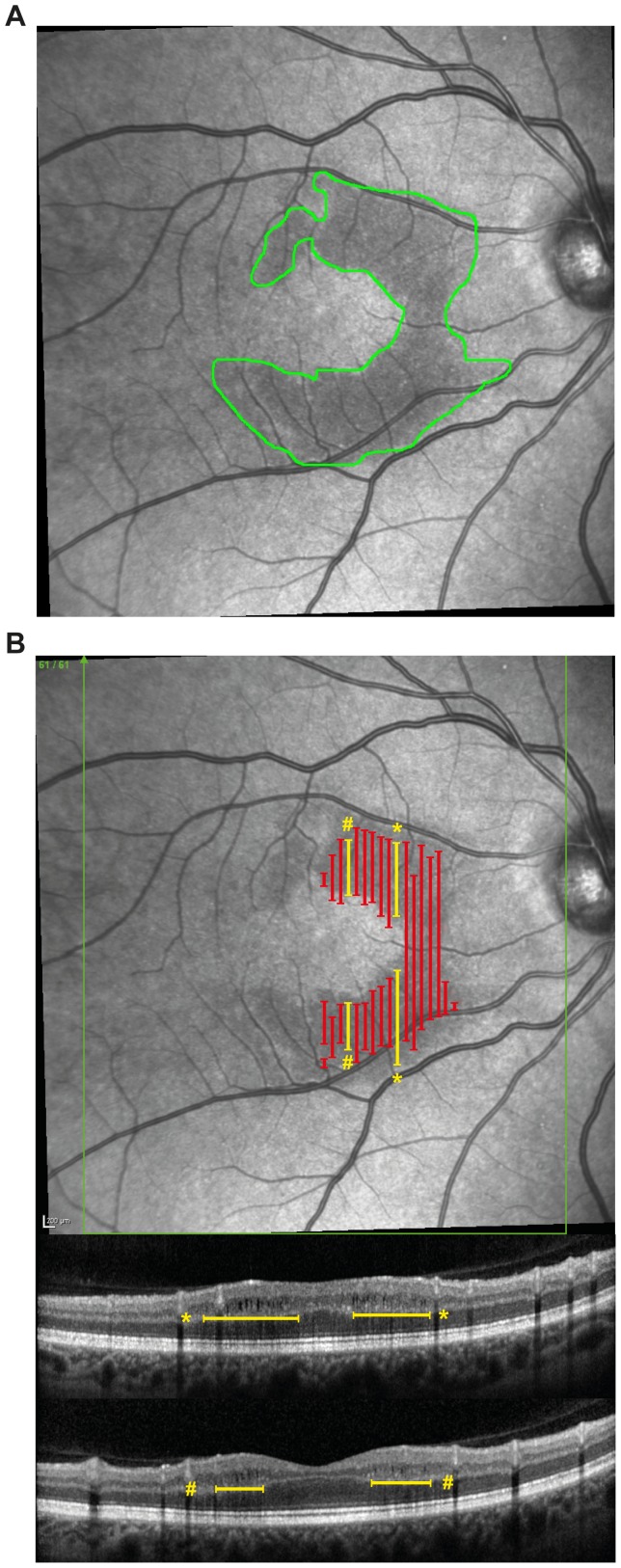
Inter-eye differences in patients with unilateral history of optic neuritis. A) Differences in inner nuclear layer (INL) thickness between affected and unaffected eyes of patients with a unilateral history of optic neuritis categorized by diagnosis. Eyes belonging to one patient are connected by lines. Lines in blue indicate eyes, which the INL of the optic neuritis eyes was thicker compared to the contralateral unaffected eyes, whereas red lines show the contrary. B) Correlation of inter-eye INL thickness differences with inter-eye macular retinal nerve fiber layer (mRNFL) thickness differences (LR: P<0.001). C) Correlation of inter-eye INL thickness differences with inter-eye ganglion cell and inner plexiform layer (GCIPL) thickness differences (LR: P = 0.011).

Based on medical records, 75 patients (11 CIS, 44 RRMS, 9 SPMS, 4 CRION, 7 NMOSD) had previously experienced an unilateral ON event (excluding patients with present MME). The mean age of this group was 38.9 years (SD = 10.5 years), which was not significantly different from the matching controls (mean = 38.1 years; SD = 11.1 years; *P* = 0.639). The gender composition in patients (47 female, 28 male) and controls (24 female, 15 male) did also not differ between both cohorts (*P* = 0.999). The results of the intra-retinal layer segmentation of patients with unilateral ON, patients with MME and the matching controls are shown in Table 4. Unsurprisingly, the ON-affected eyes showed reduced mRNFL and GCIPL thickness compared to the unaffected contralateral eyes (both *P*<0.001). INL thickness was increased in ON eyes without MME compared to contralateral NON eyes (*P*<0.001). We did not perform statistical testing of all disease cohorts separately, due to the relatively small sample size in some groups, but Figure 2A represents that – with some exceptions – ON eyes showed INL thickening in patients across all disease sub-groups. The intra-patient/inter-eye differences in INL were inversely correlated to mRNFL (R^2^ = 0.0863; *P*<0.001, linear regression, Figure 2B) and GCIPL inter-eye differences (R^2^ = 0.0431; *P* = 0.011, Figure 2C).

**Table 4:** Results of the retinal segmentation for patients with a history of unilateral ON, patients’ eyes with MME and controls.

	HC eyes	MME eyes	Non-ON eyes	ON eyes	ON vs. NON eyes P value (paired Wilcoxon)	MME vs. HCeyes P value(GEE)	MME vs. ON eyesP value (GEE)
Number of eyes	78	22	75	75			
Mean mRNFL thickness (SD)[in µm]	38.4 (3.8)	22.6 (2.5)	35.4 (5.1)	29.4 (5.8)	<0.001	<0.001	<0.001
Mean GCIPL thickness (SD)[in µm]	70.4 (5.5)	48.1 (5.7)	66.0 (6.9)	57.1 (9.0)	<0.001	<0.001	<0.001
Mean INL thickness (SD)[in µm]	33.0 (2.5)	39.0 (3.2)	33.9 (2.4)	34.5 (2.6)	<0.001	<0.001	<0.001

**Abbreviations:** MME = microcystic macular edema; ON = optic neuritis; mRNFL = macular retinal nerve fiber layer; GCIPL = ganglion cell and inner plexiform layer; INL = inner nuclear layer; SD = standard deviation; GEE = generalized estimation equation.

Eyes with MME showed a more severe reduction in mRNFL and GCIPL thickness compared to controls and also to non-MME eyes with a previous unilateral ON, while the INL thickness was increased in patients with MME-affected eyes compared to controls and eyes with a history of unilateral ON (all *P*<0.001, Table 4).

### Characteristics of MME in OCT SLO Images

The SLO images showed dark, dotted patterns in the ocular fundus in all 22 MME-affected eyes. These generally presented in a crescent shape around the fovea, which was itself recessed. Sample SLO images and their corresponding B-scans are shown in Figure 1.

To test the sensitivity and specificity of using these SLO changes to diagnose MME compared to using OCT B-scans, a second experienced operator screened all SLO images in a blinded fashion and was able to correctly classify all MME-affected eyes originally identified by OCT B-scans with no false negatives (sensitivity = 100%). An additional 27 eyes (from 17 subjects) not identified as potential MME eyes using OCT B-scans were flagged in the SLO analysis (specificity = 95.2%). Three of these eyes showed suspicious MME structures, but did not fulfill the criterion of visible microcysts on two adjacent B-scans.

### MME Size Correlates with Visual Acuity

**Figure 3:**
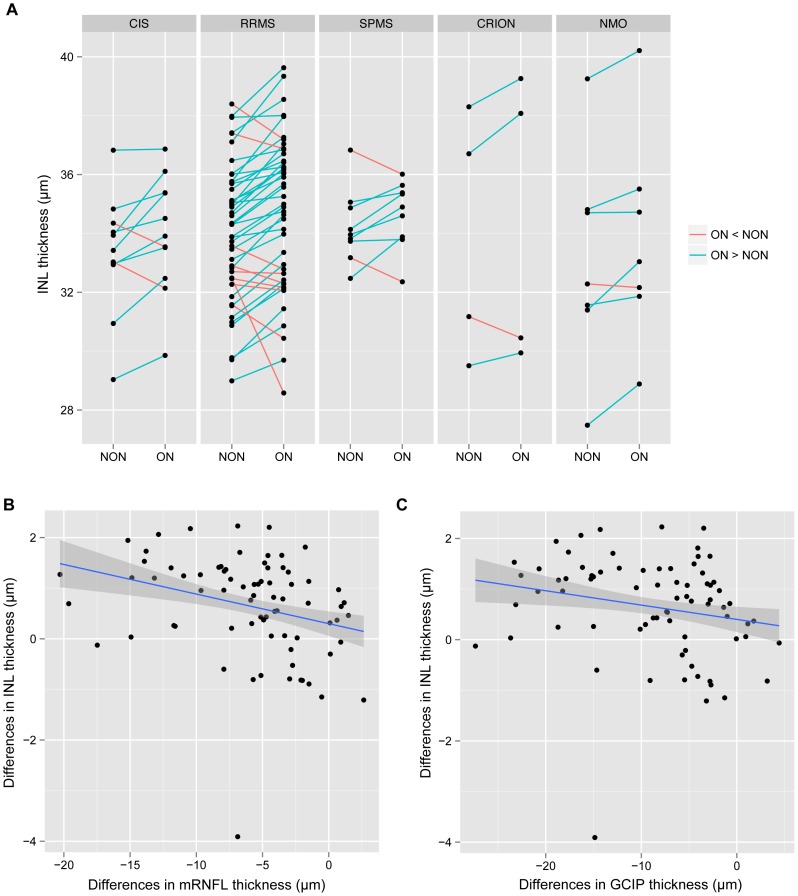
Sample quantification of SLO MME area. A) Quantification of the MME-affected area of a sample eye in a SLO image with ImageJ. B) The microcysts in OCT B-scans of the same sample eye mapped onto the SLO image. The yellow lines correspond to the spread of the macular edema in the two example B-scans at the bottom.

Alterations found in SLO images featured mostly clear contours that allowed us to quantify their area, which ranged from 1.7 to 11.1 mm^2^ (mean ± SD: 5.9±2.3 mm^2^; Figure 3A). Figure 3B maps the corresponding points of microcysts in OCT B-scans and SLO images. In GEE models, time since last ON in months (B = −0.1, SE = 0.04, *P* = 0.025) was a significant, inverse predictor for the size of MME-affected area shown in the SLO image. In other words, the shorter the time between the ON event and the OCT scan the larger the MME-affected area shown in the SLO image. Secondly, the size of the MME-affected area shown by SLO was in turn inversely correlated with visual acuity (B = −2.2, SE = 0.9, *P* = 0.012). In contrast, there was no correlation between SLO area and pRNFL thickness (*P* = 0.943) or TMV (*P* = 0.233).

## Discussion

In this study we investigated the occurrence of INL changes including MME in neuro-inflammatory diseases associated with optic neuritis. Our main findings are: a) MME was almost exclusively limited to patients with a history of ON, regardless of the underlying nosologic entity, and – with the exception of one case – only occurred in eyes previously affected by ON; b) eyes without MME but with previous ON displayed INL thickening in comparison to contralateral eyes without a history of ON; c) MME was easily detected and quantified using fundoscopic SLO images; d) the size of the affected MME-area in SLO images correlated inversely with the length of time since the last ON event and with visual acuity.

Previous studies investigating MME in MS patients have found higher ON frequencies in MME eyes compared to unaffected eyes [25], [26]. For example, in one study, 50% of MME-affected eyes of MS patients had a history of ON, while two studies found past history of ON in 100% of the MME-affected NMO eyes [27], [28]. In line with these findings, our data showed that almost all MME eyes had a history of ON, not only in NMO but also in MS and CRION patients. Overall, MME was more common in NMO and CRION than MS patients. This strongly points to a pathophysiological correlation between the development of MME and the extent of damage to the optic nerve, which would suggest that MME may result from ocular inflammation during or following ON, irrespective of the underlying nosologic entity. The finding that eyes with more severe optic neuritis were more likely to exhibit MME could indicate that the latter is an extreme manifestation of INL pathology, found in milder form as INL thickening in the majority of ON eyes. The fact that all previous studies have reported significantly reduced RNFL for MME-affected eyes, which is a common feature of ON-affected eyes, lends weight to our hypothesis [14]. Notably in this context, the only MME-affected eye in our study without a reported previous clinical ON event was that of an MS patient with long disease duration and a secondary progressive course. However, in this case, a previously recorded prolonged VEP latency and reports of visual disturbances in the past also suggest a subclinical ON event.

Importantly, previous studies did not detect general INL thickening in ON eyes from MS or NMO patients in comparison to healthy controls or non-ON eyes [26], [27], probably due to the higher inter-individual variation compared to the effect size. One study found INL thinning in primary progressive MS patients compared to healthy controls, but not in other MS types [46]. Pairwise comparison of eyes from patients with only unilateral ON and no present MME consistently showed INL thickening in the eye with previous ON compared to its ON-unaffected counterpart, and was also true for MS, CRION and NMOSD patients alike. In MS, INL thickening has been previously reported to be correlated with higher inflammatory activity and more severe disease progression [26]. Whether the former was mainly a result on the more aggressive inflammatory activity in these patients, as suggested by Saidha et al. (i.e. MS patients with higher inflammatory activity could also have more clinical or subclinical ON episodes or other ocular inflammation, such as uveitis or periphlebitis that could be mistaken for optic neuritis) or represents an additional pathology requires further investigation. In our study, thinner INL in eyes with a history of ON compared to the contralateral eyes was only found in a few cases. Although a clear explanation for these exceptions is not evident from our data, but one reason might be that subclinical ON events not documented or reported by the patient had skewed our analysis.

Histopathological data on retinal pathology in MS have been provided in a comprehensive work by Green et al. [29]. Here, the INL was identified as a prominent site of reduced neuronal cell count, inflammation and microglial activation, presumably giving rise to development of microcystic edema. However, histopathological findings of reduced neuronal cell count may not necessarily translate into reduced thickness *in vivo,* since histopathology allows only quantification of cell counts, but not of tissue layer thicknesses. The latter parameter is usually lost or altered during the fixation process. It can be assumed that specific layer properties of the INL, including the presence of bipolar cells, promote loosening of intra-layer adhesion and in some cases even cause microcystic alterations or edematous changes, such as described by Gelfand and colleagues [25]. Whether the observed INL alterations are induced by bipolar cells, Müller cells, horizontal cells, amacrine cells and/or changes of the extracellular matrix, remains to be clarified. Of the latter, Müller cells indeed play a crucial role, as their dendrites are in contact with all other retinal layers and mediate the protective or detrimental processes of other retinal cells [47]. Overall, further studies are required to bridge the gap between post-mortem analyses of human retinal tissue and *in vivo* OCT.

The potential inflammatory etiology of INL changes posited by the paper of Gelfand et. al. [25] was challenged in two reply letters which reported retinal microcystic changes in non-inflammatory diseases affecting the optic nerve, leading the authors to propose alternative underlying pathomechanisms. Abegg et al. discussed MME as a possible consequence of optic nerve degeneration in optic nerve glioma [33], whereas Barboni et al. proposed vitreous traction, resulting in a schesis stretching the INL in Leber′s hereditary optic neuropathy [32]. Both claimed that the resulting findings were identical to those reported for MS and NMO and would thus argue against disease specificity. Indeed, the included imaging data exhibited striking similarities to MME in MS, NMO and CRION. However, the small sample size and anecdotal nature of the two case reports presented in the letters hampers assessment of the proposed hypotheses [48]. Larger studies are needed to determine whether the described findings are indeed based on same mechanisms as in MS and related inflammatory diseases or are rather merely similar symptoms with different etiologies. However, histopathological data on reduced neuronal cell counts in the INL in MS [29] and our findings of INL thickening in ON eyes strongly suggest that INL changes contribute significantly to retinal pathology in optic neuritis-associated diseases. Furthermore, INL changes were recently demonstrated in a rodent model of MS in the form of microcystic disruption or repair-associated loosening of the INL layer, which supports the hypothesis of increased INL thickness in relation to optic nerve inflammation [49].

We could identify MME with high sensitivity and specificity using SLO images, a technique previously reported as promising in a case report [33]. In a blinded analysis, every MME detected on a B-scan had a clearly visible equivalent on the corresponding SLO image. Based on these findings, SLO image analysis may be superior to the more time-consuming inspection of a high number of B-scans for MME detection in daily routine. The fact that more eyes were classified as MME-affected in the blinded SLO analysis than in B-scan detection could be explained by the dynamics of microcystic changes, which might be different from the alterations found on OCT B-scans. Ultimately, with no gold standard means of confirmation (i.e. by histology), our study cannot determine whether the additional positive SLO scans were false positives or the corresponding OCT B-scans were false negatives.

The size of MME, as quantified by SLO images analysis, was inversely correlated with the length of time between prior ON and OCT examination: the shorter the interval between the clinical event of ON and OCT scan, the larger the MME-affected surface in the SLO image. Secondly, eyes with low visual acuity showed larger textural changes in SLO images than eyes with high visual acuity in patients with MME. Finally, MME-affected eyes showed significantly stronger RNFL thinning and poorer visual acuity than MME-unaffected eyes with a history of ON. These findings support the hypothesis that MME is a result of severe optic neuritis events and may contribute to a poor visual outcome following optic neuritis. However, given the cross-sectional design of our study and the in some cases long intervals between previous clinical ON, as documented or reported by patients, and MME diagnosis, these results should be interpreted with caution. Temporal dynamics of MME subsequent to ON will have to be addressed in longitudinal studies with frequent OCT scans following the clinical event.

Some important caveats deserve mention. Like the work by Gelfand et al. and Saidha et al., our study was a retrospective analysis, including assessment of previous ON events without the benefit of first-hand observation. While carries a theoretical risk of misclassification the high accordance of our findings with those in previous MME studies suggests methodological bias in our study is probably limited. Moreover, we examined a rather small cohort of CRION and NMOSD patients, which produced a very high rate of MME in these entities (56% and 15%, respectively) compared to MME prevalence in MS. The small number of these patients in our study reflect the relatively low prevalence of these diseases [34], [50], but cohort size should be increased in further studies. Although the prevalence of MME varied between the three conditions, group comparisons should be interpreted with caution due to the relatively low sample size in the NMOSD and CRION groups and the overall low frequency of MME. Moreover, we cannot exclude the possibility that some of the CRION cases may evolve into an MS or NMOSD diagnosis with a longer duration of observation. Furthermore, the quantification of MME-affected areas in SLO images includes a potential grader bias. Further verification is required to establish the MME-affected area in SLO images as an accepted marker for the degree of retinal damage caused by MME.

In summary, we show that INL thickening and MME occur in various neuro-inflammatory disorders and are strongly linked to ON events and decreased visual acuity. We also provide data that identification and quantification of MME using SLO images generated by the OCT device is easier than using the established technique of B-scans, and may lend itself to clinical application in the future. In the final instance, the causative role of ON for MME and the dynamics of INL thickening and MME formation can only be elucidated in a longitudinal study on patients with acute optic neuritis.
