# Correction: Optic Neuritis Is Associated with Inner Nuclear Layer Thickening and Microcystic Macular Edema Independently of Multiple Sclerosis

**DOI:** 10.1371/annotation/f13fb9e2-f441-4e99-bb97-79152da1e74e

**Published:** 2013-10-25

**Authors:** Falko Kaufhold, Hanna Zimmermann, Elisa Schneider, Klemens Ruprecht, Friedemann Paul, Timm Oberwahrenbrock, Alexander U. Brandt

Figures 2 and 3 were incorrectly switched. The image currently appearing as Figure 2 belongs with the title and legend for Figure 3, and the image currently appearing as Figure 3 belongs with the title and legend for Figure 2. The titles and legends themselves are in correct order. 

